# The worsening effect of paroxysmal atrial fibrillation on left ventricular function and deformation in type 2 diabetes mellitus patients: a 3.0 T cardiovascular magnetic resonance feature tracking study

**DOI:** 10.1186/s12933-024-02176-4

**Published:** 2024-03-06

**Authors:** Xue-Ming Li, Wei-Feng Yan, Ke Shi, Rui Shi, Li Jiang, Yue Gao, Chen-Yan Min, Xiao-Jing Liu, Ying-Kun Guo, Zhi-Gang Yang

**Affiliations:** 1 Department of Radiology, West China Hospital, Sichuan University, 37# Guo Xue Xiang, Chengdu, Sichuan China; 2 Laboratory of Cardiovascular Diseases, Regenerative Medicine Research Center, West China Hospital, Sichuan University, 37# Guo Xue Xiang, Chengdu, Sichuan China; 3 Department of Radiology, Key Laboratory of Birth Defects and Related Diseases of Women and Children of Ministry of Education, West China Second University Hospital, Sichuan University, 20# South Renmin Road, Chengdu, Sichuan China

**Keywords:** Type 2 diabetes mellitus, Atrial fibrillation, Cardiovascular magnetic resonance, Feature tracking, Left ventricular function

## Abstract

**Background:**

Atrial fibrillation (AF) has been linked to an increased risk of cardiovascular death, overall mortality and heart failure in patients with type 2 diabetes mellitus (T2DM). The present study investigated the additive effects of paroxysmal AF on left ventricular (LV) function and deformation in T2DM patients with or without AF using the cardiovascular magnetic resonance feature tracking (CMR-FT) technique.

**Methods:**

The present study encompassed 225 T2DM patients differentiated by the presence or absence of paroxysmal AF [T2DM(AF+) and T2DM(AF−), respectively], along with 75 age and sex matched controls, all of whom underwent CMR examination. LV function and global strains, including radial, circumferential and longitudinal peak strain (PS), as well as peak systolic and diastolic strain rates (PSSR and PDSR, respectively), were measured and compared among the groups. Multivariable linear regression analysis was used to examine the factors associated with LV global strains in patients with T2DM.

**Results:**

The T2DM(AF+) group was the oldest, had the highest LV end‑systolic volume index, lowest LV ejection fraction and estimated glomerular filtration rate compared to the control and T2DM(AF−) groups, and presented a shorter diabetes duration and lower HbA1c than the T2DM(AF−) group. LV PS-radial, PS-longitudinal and PDSR-radial declined successively from controls through the T2DM(AF−) group to the T2DM(AF+) group (all p < 0.001). Compared to the control group, LV PS-circumferential, PSSR-radial and PDSR-circumferential were decreased in the T2DM(AF+) group (all p < 0.001) but preserved in the T2DM(AF−) group. Among all clinical indices, AF was independently associated with worsening LV PS-longitudinal (β = 2.218, p < 0.001), PS-circumferential (β = 3.948, p < 0.001), PS-radial (β = − 8.40, p < 0.001), PSSR-radial and -circumferential (β = − 0.345 and 0.101, p = 0.002 and 0.014, respectively), PDSR-radial and -circumferential (β = 0.359 and − 0.14, p = 0.022 and 0.003, respectively).

**Conclusions:**

In patients with T2DM, the presence of paroxysmal AF further exacerbates LV function and deformation. Proactive prevention, regular detection and early intervention of AF could potentially benefit T2DM patients.

## Background

Type 2 diabetes mellitus (T2DM) is characterized by insulin resistance and hyperglycemia. Cardiovascular complications are the leading cause of morbidity and mortality in patients with diabetes [1], and the risk of heart failure increases 2–8 times in individuals with T2DM [2]. As the most common sustained arrhythmia worldwide, the prevalence of atrial fibrillation (AF) is expected to more than double in the next 3 decades [3]. It is characterized by rapid, disorganized excitation of the atria and irregular activation of the ventricles, and associated with a significantly high risk of cardiovascular morbidity and mortality and reduced quality of life [4, 5]. The presence of T2DM, regardless of coexisting comorbidities, increases the risk of new-onset AF [6, 7]. Changes in cardiac structure, function, and strain in T2DM patients complicated with AF are not completely understood and are challenging to evaluate. It is of paramount significance to investigate cardiac dysfunction in patients with T2DM complicated with AF before the occurrence of adverse events which may contribute to improve clinical management and reduce cardiovascular risk.

Myocardial strain, which represents the percent change in myocardial length from a relaxed to a contractile state, may be evaluated using echocardiography or cardiovascular magnetic resonance (CMR) imaging. Strain parameters, particularly global longitudinal peak strain, are known to be superior to the evaluation of left ventricular ejection fraction (LVEF) in predicting LV dysfunction and major adverse cardiac events [8]. Circumventing the echocardiographic limitations of dependence on operator experience and suboptimal acoustic windows due to the presence of bone and lung, CMR-feature tracking (CMR-FT), which quantitatively tracks myocardial features throughout the cardiac cycle on standard cine imaging, provides precise and reproducible measurements of myocardial strains [9]. It has been demonstrated to detect early LV myocardial dysfunction, including diastolic and systolic function, in patients with a variety of cardiovascular diseases [10, 11]. To the best of our knowledge, the application of this methodology to quantify myocardial strain for assessing LV myocardium abnormalities in T2DM patients with coexisting AF and no adverse events has not been reported.

Therefore, the present study evaluated the characteristics of LV volume, function and strain in T2DM patients with or without paroxysmal AF to investigate whether AF aggravated LV myocardial dysfunction in T2DM patients and determine the independent factors associated with LV global strains.

## Methods

### Study population

We retrospectively and consecutively identified 584 T2DM patients who had undergone CMR examinations in our hospital from October 2015 to December 2022. T2DM patients were diagnosed according to the recommendation of the American Diabetes Association [12]. The following exclusion criteria were used: (1) known coronary artery disease (myocardial infarction, revascularization, or coronary bypass), congenital heart disease, cardiomyopathy or severe valvular heart disease; (2) prior catheter ablation, AF at the time of CMR or uncontrolled hypertension (systolic blood pressure, SBP > 160 mmHg); (3) symptoms of heart failure, LVEF < 50% or severe renal failure (eGFR < 30 ml/ min/1.73 m^2^); and (4) incomplete clinical data, inadequate images or poor image quality affecting LV measurements. Following these criteria, a total of 225 patients were included in the study. Paroxysmal AF was defined as AF that spontaneously or therapeutically terminates within 7 days confirmed by a 12-lead resting electrocardiogram (ECG) or 24-h Holter ECG based on the contemporary clinical guidelines [13]. To evaluate the influence of AF on left ventricle, T2DM patients were further differentiated by the presence or absence of paroxysmal AF [T2DM(AF+) and T2DM(AF−) groups, respectively]. For comparison, 75 subjects who underwent CMR for health physical examination and matched with T2DM patients for age and sex were enrolled as the control group. These subjects had no (1) history of diseases that could impair cardiac function, such as hypertension, diabetes or impaired glucose tolerance, coronary heart disease, cardiomyopathy, valvular heart disease; (2) cardiovascular disease-related symptoms, such as chest pain, palpitation and dyspnea; and (3) abnormalities in electrocardiogram or cardiac abnormalities detected on CMR examination, such as decreased ejection fraction, abnormal ventricular motion, valvular stenosis or regurgitation.

The Biomedical Research Ethics Committee of West China Hospital approved this study (No. 2022-1241), which was performed in accordance with the Declaration of Helsinki. Written informed consent have been waived owing to the retrospective nature of this study.

### CMR protocol

All CMR examinations were performed using a 3.0 T MR system (TrioTim or MAGNETOM Skyra, Siemens Medical Solutions, Erlangen, Germany) with 32-channel phased array surface coils. Balanced steady-state free-precession (b-SSFP) cine images were obtained at the end of inspiratory breath-holding in a stack of standard short-axis slices and two long axes (horizontal and vertical) covering the entire left ventricle. The imaging parameters were repetition time (TR) = 2.81 ms or 3.4 ms, echo time (TE) = 1.22 ms, flip angle (FA) = 40° or 50°, field of view (FOV) = 250 × 300 mm^2^ or 340 × 285 mm^2^, matrix = 208 × 139 or 256 × 166, slice thickness = 8 mm and 25 cardiac phases. Late gadolinium enhancement (LGE) images in the entire LV short-axis stack and from the two-, three- and four-chamber views were acquired to exclude myocardial infarct 10–15 min after intravenous injection of gadolinium contrast agent (0.2 mL/kg body weight and flow rate of 2.5–3.0 mL/s). The images were obtained using a phase-sensitive inversion recovery sequence with the following parameters: TR 750/300 ms, TE 1.18/1.44 ms, FA 40°, slice thickness 8 mm, FOV 275 × 400 mm^2^ or 400 × 270 mm^2^, and matrix size = 256 × 184.

### CMR analysis

All CMR images were analyzed using dedicated software (Cvi42, v.5.11.2; Circle Cardiovascular Imaging, Calgary, Canada) by two experienced investigators who had more than three years of experience in CMR imaging and were blinded to the clinical data of the subjects.

In the Short-3D module, endocardial and epicardial borders of the left ventricle were semiautomatically outlined at the end-diastolic and end-systolic phases in the short-axis cine images. The LV functional parameters, including LVEF, end-diastolic volume (EDV), end systolic volume (ESV), stroke volume (SV) and cardiac output (CO), were assessed automatically. LV mass (LVM) was calculated by measuring the area between the endocardial and epicardial borders in each of the short-axis slices. LV papillary muscles were included in the LV volume and excluded from LVM. The body surface area (BSA) was calculated using the Mosteller formula, and the LV functional parameters and LVM were indexed for BSA. LV remodeling index was calculated as LVM/LVEDV.

**Fig. 1:**
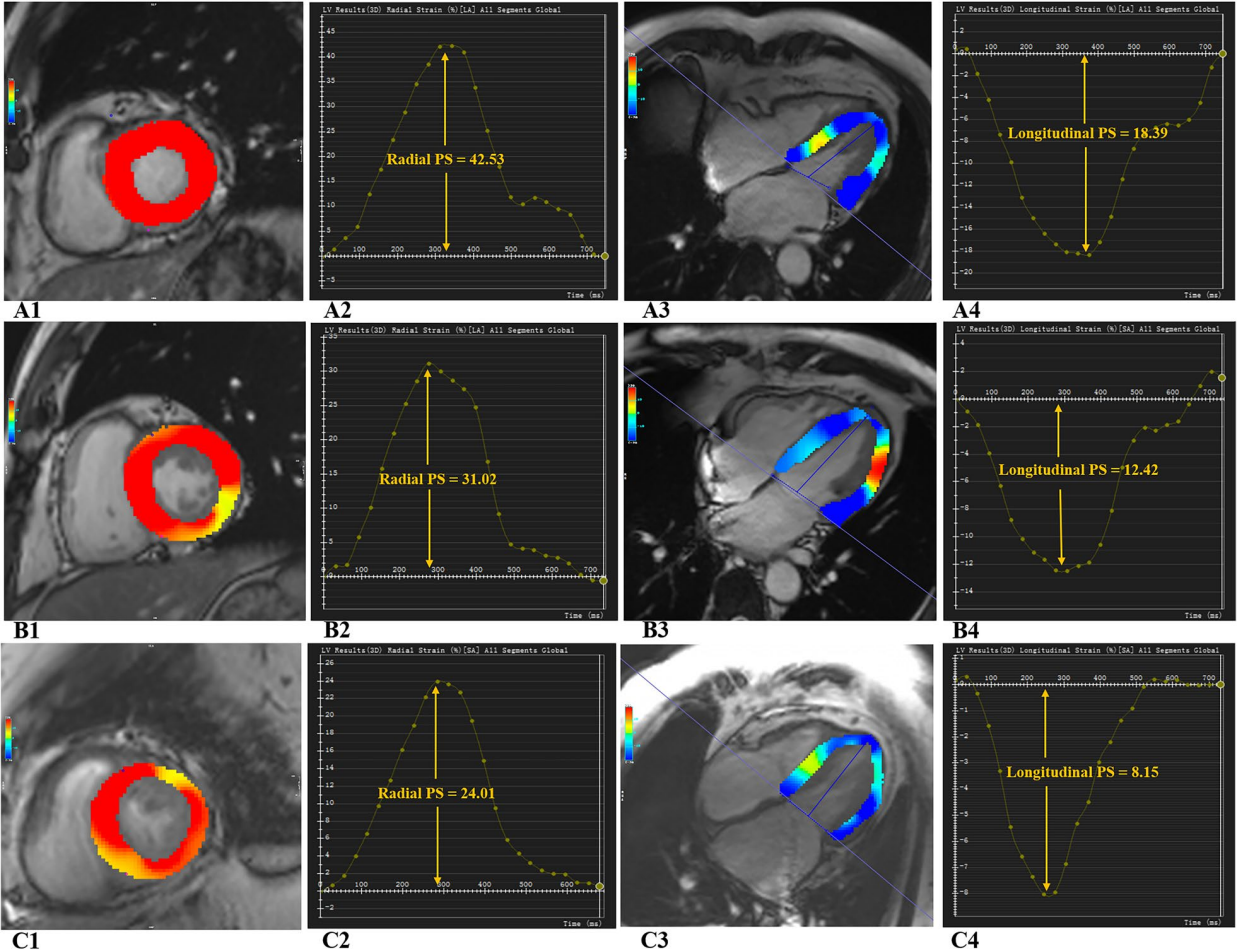
Representative cardiovascular magnetic resonance (CMR) pseudocolor images at the end‑diastole and CMR‑ derived peak strain curves. **A1**–**4**: an individual of control group; **B1**–**4**: a T2DM patient without AF; **C1**–**4**: a T2DM patient with AF. **A1**–**C1**: left ventricular (LV) pseudocolor images in short‑axis; **A2**–**C2**: LV global peak strain (PS) curve in radial direction; **A3**–**C3**: LV pseudocolor images in horizontal 4‑chamber long‑axis; **A4**–**C4**: LV global peak strain curves in longitudinal direction

For LV myocardial strain analysis, the short-axis, long-axis four- and two-chamber cine images were transferred to the tissue tracking module. The LV endocardial and epicardial borders at the end-diastolic phases (reference phase) in all series were semiautomatically delineated. The software automatically calculated the global LV strain variables (Fig. 1), including global radial, circumferential and longitudinal peak strain (PS), peak systolic strain rate (PSSR) and peak diastolic strain rate (PDSR). Strain is calculated as the percentage change in the length of myocardium and strain rate refers to the rate of myocardial deformation. PS is defined as the absolute value of maximum strain measured throughout the cardiac cycle, PSSR defined as the absolute value of the maximum strain rate from end diastole to end systole, and PDSR defined as the absolute value of the maximum strain rate from end systole to end diastole. During the systolic phase, the radial strain had a positive value due to myocardium thickening, and the circumferential and longitudinal strains had negative values because the myocardium shortened.

### Reproducibility analysis

To assess the intra-observer variability, one investigator measured the LV global strain parameters in 60 randomly selected subjects (40 T2DM patients and 20 healthy controls) twice after one month. A second investigator, who was blinded to the results of the first investigator and clinical data, reevaluated the measurements to determine the interobserver variability.

### Statistical analysis

All statistical analyses were performed using SPSS (version 23.0, IBM SPSS Inc., Armonk, New York, USA). Continuous data are presented as the means ± standard deviation (SD) or medians with interquartile range (IQR), and categorical variables are presented as n (%). Parameters among the T2DM(AF−), T2DM(AF+) and control groups were compared using one-way analysis of variance (one-way ANOVA) with post hoc Bonferroni correction (normally distributed variables) or Kruskal‒Wallis tests (nonparametric variables), as appropriate. The diabetes duration and HbA1c level between the patient groups were compared using the Mann–Whitney U test. Categorical variables were compared using the chi-squared or Fisher’s exact test, as appropriate. Univariable linear regression analyses were performed to demonstrate the relationship between candidate factors and LV global strain parameters. Variables with a value of p < 0.1 in univariable analysis and no collinearity as well as AF, age and sex were included in stepwise multivariable linear regression analyses to determine the independent determinants of LV global strains. A variance inflation factor (VIF) of 5 was set to avoid multicollinearity between the univariable parameters. Intraclass correlation coefficients (ICCs) were evaluated to determine the inter- and intra-observer variability in strain measurement. A two-sided p < 0.05 was considered statistically significant.

## Results

### Baseline characteristics

Of the 225 T2DM patients, 182 patients were in the T2DM(AF−) group (102 [56.0%] males, mean age 58.2 ± 9.1 years), and 43 patients were in the T2DM(AF+) group (19 [44.2%] males, mean age 67.1 ± 10.7 years). The main baseline characteristics of the patient groups and controls are presented in Table 1. Both patient groups showed higher body mass index (BMI), SBP and fasting blood glucose than controls (all p < 0.05). Patients with T2DM(AF+) were older than the control and T2DM(AF−) groups (all p < 0.001) but had a shorter diabetes duration, lower HbA1c and eGFR levels than patients without AF (all p < 0.05). Patients with T2DM(AF+) had lower use of oral and insulin antidiabetic therapy (all p < 0.05) but higher use of β‑blockers than the T2DM(AF−) group (p = 0.011).

**Table 1:** Baseline characteristics of the study cohort

	Controls (n = 75)	T2DM(AF−) (n = 182)	T2DM(AF+) (n = 43)
Male, n (%)	38 (50.7)	102 (56.0)	19 (44.2)
Age (years)	57.0 ± 11.1	58.2 ± 9.1	67.1 ± 10.7*^†^
BMI (kg/m^2^)	22.97 ± 2.49	24.35 ± 3.47*	25.16 ± 4.06*
BSA (kg/m^2^)	1.71 ± 0.18	1.71 ± 0.16	1.68 ± 0.17
Heart rate (beats/min)	71.2 ± 11.4	72.6 (65.2, 81.9)	73.2 ± 14.9
SBP (mmHg)	120 (106, 125)	127 (119, 140)*	128 (120, 143)*
DBP (mmHg)	74.9 ± 8.5	80.2 ± 10.9*	78.6 ± 12.8
Diabetes duration (years)	NA	6 (2.8, 12)	1 (0.5, 4.5)^†^
AF duration (months)	NA	NA	2 (1,12)
CHA(2)DS(2)-VASc score	NA	NA	4 (2,5)
*Laboratory data*			
FBG (mmol/L)	5.12 (4.78, 5.73)	7.30 (6.10, 8.95)*	8.32 (6.44, 11.78)*
HbA1c (%)	NA	7.01 (6.40, 8.22)	6.45 (6.20, 7.3)^†^
Triglycerides (mmol/L)	1.38 ± 0.57	1.67 ± 1.23	1.61 ± 1.82
Total cholesterol (mmol/L)	4.51 ± 0.78	4.35 ± 0.96	3.72 ± 1.03*^†^
HDL (mmol/L)	1.36 ± 0.37	1.27 ± 0.40	1.16 ± 0.38*
LDL (mmol/L)	2.66 ± 0.67	2.59 ± 0.85	1.97 ± 0.77*^†^
eGFR (mL/min/1.73 m^2^)	88.57 ± 19.06	90.77 ± 18.93	73.69 ± 28.39*^†^
*Medications, n (%)*			
Statin	NA	49 (26.9)	5 (11.4)^†^
Biguanides	NA	108 (59.3)	12 (37.3)^†^
Sulfonylureas	NA	45 (24.7)	9 (20.5)
α-Glucosidase inhibitor	NA	74 (40.7)	5 (11.4)^†^
Insulin	NA	60 (33.0)	6 (13.6)^†^
ACEI/ARB	NA	46 (25.3)	15 (34.9)
β-blocker	NA	19 (10.5)	12 (27.9)^†^
Calcium channel blocker	NA	54 (29.7)	13 (30.2)
Ia/Ic antiarrhythmics	NA	NA	4 (9.3)

The values are the mean ± SD, Numbers in the brackets are percentages. T2DM, type 2 diabetes mellitus; AF, atrial fibrillation; BMI, body mass index; BSA, body surface area; SBP, systolic blood pressure; DBP, diastolic blood pressure; FBG, fasting blood glucose; HbA1c, glycated hemoglobin; HDL, high-density lipoprotein cholesterol; LDL, low-density lipoprotein cholesterol; eGFR, estimated glomerular filtration rate; ACEI, angiotensin converting enzyme inhibitor; ARB, angiotensin II receptor blocker

CHA(2)DS(2)-VASc score indicate a measure of the risk of stroke in which congestive heart failure, hypertension, diabetes mellitus, vascular disease, an age of 65–74 years and female are each assigned 1 point, and an age of ≥ 75 years and previous stroke or transient ischemic attack are assigned 2 point [13]

^*^p < 0.05 versus controls

^†^P < 0.05 versus T2DM(AF−) group

### Comparison of LV functional and strain parameters

The CMR-measured LV parameters for the patient groups and healthy controls are shown in Table 2. Compared to the controls, the T2DM(AF−) and T2DM(AF+) groups showed a significantly larger LVMI (p < 0.001 and p = 0.012, respectively) and LV remodeling index (p = 0.001 and = 0.036, respectively), but these parameters were not significantly different between the patient groups. The LVESVI and LVEF in the T2DM(AF+) group were significantly higher and lower, respectively, than the T2DM(AF−) group (p = 0.021 and p < 0.001, respectively) and controls (p = 0.014 and p = 0.001, respectively). The LVEDVI, LVSVI and cardiac index were not significantly different among the groups (all p > 0.05).

**Table 2:** CMR findings between controls, T2DM(AF−) group and T2DM(AF+) group

	Controls	T2DM(AF−)	T2DM(AF+)	P value
*LV geometry and function*				
LVEDVI (ml/m^2^)	72.60 ± 11.67	74.05 ± 15.04	77.18 ± 19.71	0.787
LVESVI (ml/m^2^)	26.01 ± 6.60	26.75 ± 8.62	31.50 ± 11.10*^†^	0.014
LVSVI (ml/m^2^)	46.59 ± 8.27	47.31 ± 9.89	45.68 ± 11.75	0.674
LVCI (L/min/m^2^)	3.32 ± 0.81	3.49 ± 0.79	3.15 ± 1.27	0.28
LVEF (%)	64.31 ± 6.16	64.26 ± 7.26	59.03 ± 7.13*^†^	< 0.001
LVMI (g/m^2^)	41.46 ± 8.56	48.40 ± 12.40*	49.29 ± 14.91*	< 0.001
LV remodeling index (g/mL)	0.58 ± 0.12	0.67 ± 0.18*	0.67 ± 0.21*	0.001
*Myocardial strain*				
PS, %				
Radial	36.55 ± 7.25	33.29 ± 8.52*	26.66 ± 9.31*^†^	< 0.001
Circumferential	− 20.84 ± 2.61	− 19.94 ± 2.75	− 16.46 ± 4.50*^†^	< 0.001
Longitudinal	− 14.87 ± 2.44	− 12.91 ± 2.94*	− 10.14 ± 3.60*^†^	< 0.001
PSSR, 1/s				
Radial	2.02 (1.65, 2.32)	1.75 (1.52, 2.31)	1.43 (1.16, 1.92)*^†^	< 0.001
Circumferential	− 1.06 (− 1.20, − 0.89)	− 1.00 (− 1.15, − 0.90)	− 0.99 (− 1.12, − 0.70)	0.366
Longitudinal	− 0.78 (− 0.88, − 0.70)	− 0.73 (− 0.86, − 0.63)*	− 0.69 (− 0.86, − 0.57)*	0.008
PDSR, 1/s				
Radial	− 2.29 (− 3.02, − 1.88)	− 2.04 (− 2.62, − 1.72)*	− 1.77 (− 2.17, − 1.28)*^†^	< 0.001
Circumferential	1.23 ± 0.29	1.14 ± 0.24	1.07 ± 0.73*^†^	< 0.001
Longitudinal	0.87 (0.71, 1.02)	0.78 (0.66, 0.92)*	0.67 (0.51, 0.98)*	0.001

T2DM, type 2 diabetes mellitus; AF, atrial fibrillation; LV, left ventricular; M, mass; EDV, end diastolic volume; ESV, end systolic volume; SV, stroke volume; I, indexed to BSA; EF, ejection fraction; PS, peak strain; PSSR, peak systolic strain rate; PDSR, peak diastolic strain rate

^*^p < 0.05 versus controls

^†^p < 0.05 versus T2DM(AF−) group

The LV PS-radial, PS-longitudinal and PDSR-radial declined significantly from controls through the T2DM(AF−) group to the T2DM(AF+) group (all p < 0.001). Compared to the control group, the LV PS-circumferential, PSSR-radial and PDSR-circumferential were decreased in the T2DM(AF+) group (all p < 0.001) but preserved in the T2DM(AF−) group. The PSSR-longitudinal and PDSR-longitudinal were reduced in both patient groups, but these parameters were not significantly different between these groups.

### Determinants of LV strains

**Fig. 2:**
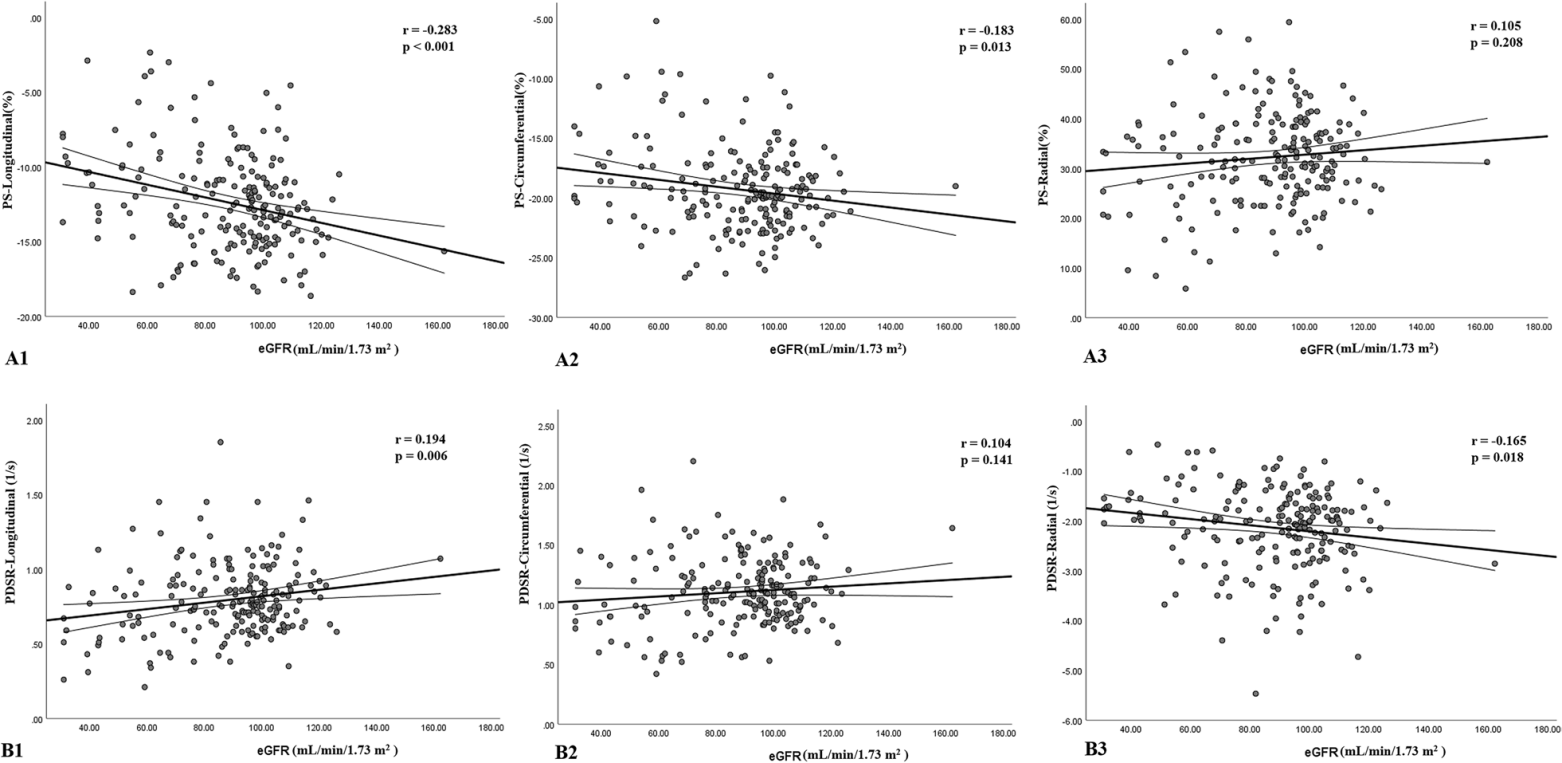
Linear regression analysis between LV peak strain (**A1**–**A3**) or PDSR (**B1**–**B3**) and eGFR in T2DM patients. eGFR, estimated glomerular filtration rate; PS, peak strain; PDSR, peak diastolic strain rate

After univariable linear regression analysis (Table 3), AF was significantly associated with all three directions of LV global PS (all p < 0.001). eGFR was significantly associated with LV PS-longitudinal and PS-circumferential (p < 0.001 and = 0.013, respectively) (Fig. 2A). The univariable linear regression analyses for LV PSSR and PDSR are shown in Table 4. AF was significantly associated with LV PSSR-radial and all three directions of PDSR (all p < 0.1), and eGFR was associated with LV PDSR-radial and PDSR-longitudinal (all p < 0.05) (Fig. 2B).

**Table 3:** Univariable and multivariable analysis between the magnitude of LV peak strain and clinical indices in T2DM patients

	PS-longitudinal				PS-circumferential				PS-radial			
	Univariable		Multivariable		Univariable		Multivariable		Univariable		Multivariable	
	B	p	B^a^	p	B	p	B^a^	p	B	p	B^a^	p
AF	2.804	< 0.001	2.218	< 0.001	3.409	< 0.001	3.948	< 0.001	− 6.243	< 0.001	− 8.40	< 0.001
Age (years)	0.036	0.099	− 0.105	0.188	0.013	0.584	− 0.089	0.203	0.041	0.504	0.138	0.040
Sex (male = 1)	1.198	0.006	1.378	0.003	1.042	0.022	1.495	0.001	− 3.249	0.007	− 3.946	0.002
BMI (kg/m^2)^	0.069	0.275			0.096	0.136			− 0.015	0.933		
HR (beats/min)	0.013	0.426			0.019	0.318			− 0.026	0.613		
SBP (per 10 mmHg)	0.021	0.086	0.111	0.103	0.004	0.756			− 0.013	0.707		
FBP (mmol/L)	− 0.004	0.939			− 0.054	0.382			− 0.007	0.967		
HbA1c (%)	− 0.073	0.566			− 0.011	0.935			− 0.175	0.613		
Triglycerides (mmol/L)	0.179	0.296			0.010	0.954			0.032	0.947		
Total cholesterol (mmol/L)	− 0.340	0.095	0.025	0.731	− 0.390	0.066	0.012	0.866	0.755	0.150		
HDL (mmol/L)	− 1.752	0.002	− 0.950	> 0.999	− 1.129	0.06	− 0.046	0.509	0.729	0.650		
LDL (mmol/L)	− 0.425	0.111			− 0.629	0.023	− 0.035	0.607	1.252	0.091	0.059	0.406
eGFR (mL/min/1.73 m^2^)	− 0.043	< 0.001	− 0.031	0.004	− 0.028	0.013	− 0.050	0.465	0.038	0.208		
Smoking	0.977	0.046	− 0.070	0.349	0.124	0.02	0.092	0.218	− 4.433	0.002	− 0.091	0.243
Diabetic duration (years)	− 0.028	0.454			− 0.092	0.019	− 0.064	0.342	0.175	0.095	− 0.017	0.826

T2DM, type 2 diabetes mellitus; AF, atrial fibrillation; BMI, body mass index; SBP, systolic blood pressure; FBG, fasting blood glucose; HbA1c, glycated hemoglobin; HDL, high-density lipoprotein cholesterol; LDL, low-density lipoprotein cholesterol; eGFR, estimated glomerular filtration rate; LV, left ventricular; PS, peak strain

^a^Variables with P < 0.1 in the univariable analysis as well as AF, age and sex were included in the multivariable analysis

**Table 4:** Univariate and multivariate analysis between the magnitude of LV peak systolic or diastolic strain rate and clinical indices in T2DM patients

	PSSR-longitudinal				PSSR-circumferential				PSSR-radial			
	Univariable		Multivariable		Univariable		Multivariable		Univariable		Multivariable	
	B	p	B^a^	p	B	p	B^a^	p	B	p	B^a^	p
AF	0.028	0.402	0.069	0.071	0.06	0.124	0.101	0.014	− 0.352	0.003	− 0.345	0.002
Age (years)	0.001	0.39	− 0.033	0.650	0.002	0.248	− 0.022	0.753	− 0.007	0.143	0.021	0.755
Sex (male = 1)	0.007	0.771	− 0.019	0.774	0.001	0.732	− 0.050	0.435	0.008	0.539	− 0.068	0.336
BMI (kg/m^2)^	0.001	0.752			0.001	0.732			0.008	0.539		
HR (beats/min)	− 0.005	< 0.001	− 0.005	< 0.001	− 0.008	< 0.001	− 0.009	< 0.001	0.024	< 0.001	0.013	0.001
SBP (per 10 mmHg)	0.001	0.304			0.001	0.439			− 0.006	0.016	− 0.063	0.011
FBP (mmol/L)	− 0.002	0.637			− 0.003	0.413			0.005	0.671		
HbA1c (%)	0.004	0.584			0.001	0.874			− 0.011	0.662		
Triglycerides (mmol/L)	− 0.006	0.57			− 0.016	0.174			0.039	0.260		
Total cholesterol (mmol/L)	0.001	0.918			0.012	0.363			− 0.024	0.555		
HDL (mmol/L)	0.015	0.643			0.071	0.065	0.081	0.207	− 0.264	0.023	− 0.253	0.021
LDL (mmol/L)	0.005	0.759			0.017	0.344			0.006	0.120		
eGFR (mL/min/1.73 m^2^)	− 0.001	0.224			− 0.028	0.955			0.001	0.625		
Smoking	− 0.021	0.473			− 0.033	0.334			− 0.057	0.582		
diabetic duration (years)	0.002	0.472			< 0.001	0.961			< 0.001	0.978		

	PDSR-longitudinal				PDSR-circumferential				PDSR-radial			
	Univariable		Multivariable		Univariable		Multivariable		Univariable		Multivariable	
	B	p	B^a^	p	B	p	B^a^	p	B	p	B^a^	p
AF	− 0.073	0.086	− 0.062	0.419	− 0.163	0.001	− 0.14	0.003	0.43	0.002	0.359	0.022
Age (years)	− 0.001	0.374	0.004	0.032	− 0.004	0.06	0.037	0.609	0.007	0.173	− 0.054	0.487
Sex (male = 1)	− 0.037	0.249	− 0.092	0.193	− 0.038	0.315	− 0.071	0.311	0.218	0.043	0.261	0.024
BMI (kg/m^2)^	0.001	0.775			− 0.009	0.106			0.002	0.157		
HR (beats/min)	0.006	< 0.001	0.005	0.001	0.007	< 0.001	0.006	< 0.001	− 0.006	0.193	− 0.023	0.748
SBP (per 10 mmHg)	− 0.001	0.311			− 0.001	0.553			0.005	0.11	0.068	0.039
FBP (mmol/L)	0.002	0.65			0.004	0.394			0.005	0.739		
HbA1c (%)	− 0.001	0.898			0.002	0.847			0.002	0.939		
Triglycerides (mmol/L)	− 0.022	0.085	− 0.115	0.103	− 0.014	0.333			0.009	0.824		
Total cholesterol (mmol/L)	0.016	0.298			0.028	0.108			− 0.093	0.064	0.115	0.436
HDL (mmol/L)	0.046	0.275			0.107	0.031	0.092	0.048	− 0.121	0.394		
LDL (mmol/L)	0.033	0.094	0.101	0.162	0.038	0.096	0.039	0.579	− 0.159	0.015	− 0.155	0.029
eGFR (mL/min/1.73 m^2^)	0.002	0.006	0.003	0.001	0.001	0.141			− 0.006	0.018	− 0.042	0.595
Smoking	− 0.005	0.892			0.015	0.742			0.217	0.082	− 0.017	0.835
Diabetic duration (years)	0.004	0.094	0.106	0.144	0.004	0.249			− 0.012	0.189		

T2DM, type 2 diabetes mellitus; AF, atrial fibrillation; BMI, body mass index; SBP, systolic blood pressure; FBG, fasting blood glucose; HbA1c, glycated hemoglobin; HDL, high-density lipoprotein cholesterol; LDL, low-density lipoprotein cholesterol; eGFR, estimated glomerular filtration rate; LV, left ventricular; PSSR, peak systolic strain rate; PDSR, peak diastolic strain rate

^a^Variables with P < 0.1 in the univariable analysis as well as AF, age and sex were included in the multivariable analysis

Multivariable linear regression analyses adjusting for confounders revealed that AF were independently associated with LV PS-longitudinal, -circumferential and -radial (β = 2.218, 3.948 and − 8.40, all p < 0.001), and eGFR was associated with LV PS-longitudinal (β = − 0.031, p = 0.004). AF was independently associated with PSSR-radial and -circumferential (β = − 0.345 and 0.101, p = 0.002 and 0.014, respectively), PDSR-radial and -circumferential (β = 0.359 and − 0.14, p = 0.022 and 0.003, respectively), but not with PSSR-longitudinal or PDSR-longitudinal. In addition, eGFR was independently associated with LV PDSR-longitudinal (β = 0.003, p = 0.001). Detailed information is shown in Tables 3 and 4.

### Intra- and interobserver variability

The measured CMR parameters were highly reproducible on an intra- and inter-observer level (Table 5), which were reflected in the ICC (0.872–0.975) for the intra-observer reproducibility and (0.812–0.942) for the inter-observer reproducibility.

**Table 5:** Intra-and inter-observer variability of LV global strain and strain rate

	Intra-observer		Inter-observer	
	ICC	95%CI	ICC	95%CI
*LV PS*				
Radial	0.936	0.857–0.972	0.902	0.831–0.953
Circumferential	0.975	0.948–0.986	0.931	0.861–0.972
Longitudinal	0.973	0.918–0.975	0.936	0.861–0.965
*LV PSSR*				
Radial	0.872	0.781–0.973	0.842	0.725–0.923
Circumferential	0.973	0.949–0.983	0.869	0.747–0.942
Longitudinal	0.891	0.769–0.953	0.812	0.7521–0.913
*LV PDSR*				
Radial	0.931	0.872–0.959	0.812	0.695–0.892
Circumferential	0.958	0.947–0.979	0.942	0.858–0.974
Longitudinal	0.925	0.837–0.972	0.819	0.692–0.912

LV, left ventricular; PS, peak strain; PSSR, peak systolic strain rate; PSDR, peak diastolic strain rate. ICC, intraclass correlation coefficient; CI, confidence interval

## Discussion

The present study investigated the combined effects of paroxysmal AF on CMR-derived LV functional and strain parameters in T2DM patients with no coexistent cardiovascular events and examined the independent predictors of LV dysfunction. Our study showed that (1) T2DM patients demonstrated adverse LV remodeling and subclinical LV systolic and diastolic dysfunction even in the absence of AF. (2) When AF was present, T2DM patients showed a significant decrease in LVEF and a more severe reduction in systolic and diastolic strain indices than T2DM patients without AF and normal controls. (3) After adjusting for confounding factors, AF and eGFR were independent predictors of reduced LV systolic and diastolic function in T2DM patients.

Diabetic cardiomyopathy is a common complication of diabetes and the most common cause of mortality. Several previous studies reported adverse LV remodeling and dysfunction in diabetes, which was demonstrated by increased LVMI and diastolic dysfunction, with or without systolic dysfunction [14–16]. The role of CMR-FT in the detection of early cardiac dysfunction in diabetes has gained attention in recent years. Using the CMR-FT method, our results showed that absolute values of the LV radial and longitudinal PS and PDSR as well as longitudinal PSSR were significantly reduced in the T2DM patients without AF, but they had comparable LVEF to the controls. These results showed that this CMR measured LV dysfunction detected subclinical contractile and diastolic dysfunction in T2DM; which further confirmed that the CMR-FT derived parameters are more sensitive in identifying subclinical alterations in LV function. The precise underlying mechanisms of T2DM on LV systolic and diastolic dysfunction are not fully understood but are likely multifactorial and may be explained as follows. Hyperglycemia, metabolism-related disorders and insulin resistance trigger oxidative stress, an inflammatory response and thrombosis, which lead to calcium handling disturbance, excitation–contraction coupling disorders, myocardial interstitial fibrosis and microvascular abnormalities [17, 18].

Despite being a very frequent scenario in daily clinical practice, descriptions of CMR-measured LV myocardial abnormalities in AF patients are limited in the literature. A previous study showed that T1 mapping detected LV myocardial abnormalities and correlated well with myocardial fibrosis in patients with AF and combined cardiovascular diseases [19, 20]. In AF patients without coexisting cardiovascular disease, a series of studies showed subclinical LV dysfunction manifested as impaired strain indices [21, 22], diffusion myocardial fibrosis (higher native T1 [21] and lower post-contrast T1 [23]), and reduced myocardial energetics using ^31^P magnetic resonance spectroscopy [24]. Although the mean LVEF of all T2DM patients enrolled was greater than 50% in our study, we found that patients with T2DM(AF+) showed a significantly higher LVESVI and lower LVEF than normal controls and patients with T2DM(AF−). This result revealed that the damage due to AF in T2DM patients already existed even when the LVEF was at a relatively normal level. Moreover, the T2DM(AF+) group showed a more remarkedly decreased LV global PS and PDSR in three directions, and PSSR-radial and longitudinal, and some of these parameters were lower than T2DM patients without AF. The mechanism for cardiac impairment in AF may be hemodynamic or nonhemodynamic. The deleterious hemodynamic effect of AF is explained by several pathophysiological mechanisms, such as loss of effective atrial contraction, shortening LV diastolic filling by tachycardia, or heart rhythm irregularity causing neurohumoral activation [25]. The nonhemodynamic deterioration effect was functional and structural alterations at the cellular level, such as reduced Ca^2+^ homeostasis (indicating negative inotropic effects), prolonged action potential duration and decreased sarcomere regularity (impaired cardiac contractility) [26]. In addition, myocardial perfusion is impaired in patients with AF, and it is related to LV systolic and diastolic dysfunction [22].

When T2DM and AF are combined, patients have a significantly higher risk for cardiovascular death, overall mortality and heart failure [27, 28], but available data on the AF-related influence on LV function in T2DM patients are scarce. Although there was a shorter diabetic duration, less insulin using and lower HbA1c level, the cardiac dysfunction was more severe in the T2DM(AF+) group than the T2DM(AF−) group. Therefore, we speculate that the myocardial impairment due to diabetes in T2DM(AF+) is lower, and the more severe cardiac dysfunction in this patient group may be due to the superposition effect of AF. As explained above, T2DM and AF each impair the heart in their own ways. When T2DM is comorbid with AF, these superimposed factors in the myocardium may be amplified and promote the aggravation of myocardial contractility and relaxation impairment. Effective treatment aimed at preventing the occurrence and timely management of AF in patients with diabetes may improve survival outcomes, in which sodium dependent glucose cotransporter 2 inhibitors (SGLT2i) significantly reduced the incidence of AF [29] and were associated with a lower risk of hospitalization for heart failure [30]. Several studies revealed that rhythm control in AF patients had reduced mortality, better quality of life, and reverse remodeling with improved LV function and cardiovascular outcomes [20, 31, 32]. Therefore, once T2DM is diagnosed, clinicians and patients should pay attention to the prevention, detection and intervention of AF.

In addition, we identified that patients with T2DM(AF+) had lower eGFR level than patients without AF and controls. Several previous studies showed that kidney function (lower eGFR and proteinuria) independently contributed to a higher risk of incident AF, and incident AF was also independently associated with a higher risk of developing end-stage renal disease and the development and progression of chronic kidney disease at earlier stages, furthermore, both of these conditions were associated with a higher risk of heart failure [33–35]. As a cross-sectional study, we could not explore the casualty between kidney dysfunction and the occurrence of AF. However, our results revealed that eGFR was independently associated with the magnitude of LV longitudinal PS and PDSR in T2DM patients. We inferred that renal dysfunction had an adverse effect on LV myocardial deformation and cardiac function in T2DM patients. Various mechanisms, including hemodynamic disturbance, neuroendocrine system activation, oxidative stress, anemia and metabolism remodeling, lead to myocardial injury in these patients and result in adverse cardiac outcomes during the process of chronic kidney dysfunction [36, 37]. Therefore, it is of great importance to monitor and intervene in renal dysfunction in patients with T2DM, especially in those with coexisting AF.

### Limitations

There are some limitations in the present study. First, this study was retrospective in nature, and the sample size of patients with paroxysmal AF suitable for the present study was small. Therefore, potential selection bias may be present, and the results require further validation. Second, this study was a cross-sectional study that could not establish causality between T2DM and AF, and future studies with long-term follow-up are needed to comprehensively investigate the evolution of cardiac remodeling and function over time with AF initiation and progression. Finally, we did not take into account the duration and severity of AF, which could potentially influence LV myocardial remodeling. We hope to investigate these factors in our forthcoming research efforts.

## Conclusions

AF had an additive deleterious effect on LV function and global strains in patients with T2DM. It should receive more attention when patients are diagnosed with T2DM, and proactive prevention, regular detection and early intervention of AF may be beneficial for T2DM patients.

## Data Availability

The datasets used in the current study are available from the corresponding author on reasonable request.

## Abbreviations

AF: Atrial fibrillation
T2DM: Type 2 diabetes mellitus
LV: Left ventricular
EF: Ejection fraction
CMR: Cardiovascular magnetic resonance
CMR-FT: CMR feature tracking
EDV: End-diastolic volume
ESV: End-diastolic volume
LVMI: LV mass index
PS: Peak strain
PSSR: Peak systolic strain rate
PDSR: Peak diastolic strain rate
